# Supplementary material for: Dynamics of Pd Subsurface Hydride Formation and Their Impact on the Selectivity Control for Selective Butadiene Hydrogenation Reaction
Source: Nanomaterials (Basel). 2023 Mar 19;13(6):1099. doi: 10.3390/nano13061099 (PMC10058484; doi:10.3390/nano13061099)
Supplement: Supplementary file 1 [file nanomaterials-13-01099-s001.zip › nanomaterials-2264753-supplementary.pdf]

nanomaterials

Supplementary Materials

Dynamics of Pd Subsurface Hydride Formation and Their Impact on the Selectivity Control for Selective Butadiene Hydrogenation Reaction

Esther Asedegbega-Nieto [1,*], Ana Iglesias-Juez [2], Marco Di Michiel [3], Marcos Fernandez-Garcia [2], Inmaculada Rodriguez-Ramos [2] and Antonio Guerrero-Ruiz [1,*]

1 Dpto. Química Inorgánica y Técnica, Facultad de Ciencias, UNED, Av. de Esparta s/n, 28232 Las Rozas, Madrid, Spain
2 Instituto de Catálisis y Petroleoquímica, CSIC, c/Marie Curie No. 2, Cantoblanco, 28049 Madrid, Spain.
3 ESRF—The European Synchrotron, 71 Avenue des Martyrs, 38000 Grenoble, France
* Correspondence: easedegbega@ccia.uned.es; aguerrero@ccia.uned.es

Abstract: Structure-sensitive catalyzed reactions can be influenced by a number

1.1. HEXRD of Pd (111) for sample 1PdG……………………………………………….…….3
1.2. HEXRD of Pd (111) for sample 1PdGOE……………………………………………..……..4
1.3. HEXRD of Pd (111) for sample 1PdGONE…………………………………………….…..5
1.4. HEXRD of Pd (111) for sample 1PdGOE fast……………………………………….…7
1.5. HEXRD of Pd (111) for sample 1PdGONE fast………………………………..…11
1.6. HEXRD of Pd (111) for sample 2PdG fast……………………………………………15
1.7. TEM images and particle size histogram of 1PdGOE……………………………19
1.8. TEM images and particle size histogram of 1PdGONE…………………………..20
1.9. TEM images and particle size histogram of 2PdG…………………………………21
1.10. TEM images and particle size histogram of 1PdG…………………………………22
1.11. Kinetics of transformation of PdH_x to Pd……………………………………………..23

1. Supplementary Results and Discussion

1.1. HEXRD of Pd (111) for sample 1PdG

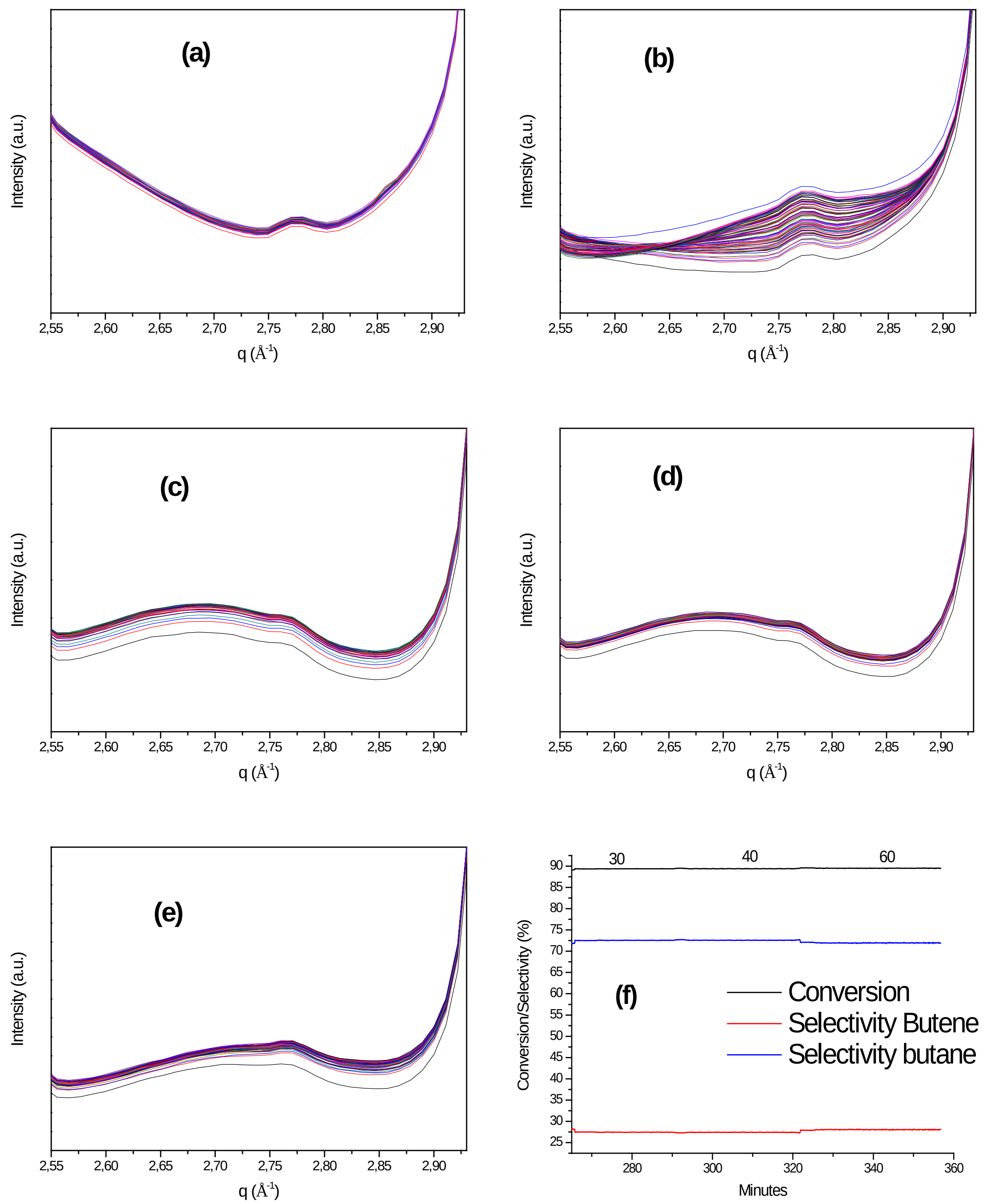

Figure S1. HEXRD of Pd (111) during temperature programmed reduction (TPR) (a), upon cooling (b), during reaction (temporal evolution when switching from H_2 to reaction mixture) at different temperatures 30 (c), 40 (d) and 60 °C (e) and conversion/selectivity (f) for sample 1PdG.

These results belong to the first set of experiments. In Figure S1 (a) the apparition of a small peak at about 2.8 $Å^{-1}$ owing to the presence of reduced Pd is observed. During cooling down to RT (b) there is no change in the position of this peak, indicating that PdH_x phase is not formed. Changes are also absent throughout the reaction at temperatures

between 30 – 60 °C ((c), (d) and (e)). From (f) we observe that high conversion values are obtained while selectivity towards the complete hydrogenated product, butane, is favored.

1.2. HEXRD of Pd (111) for sample 1PdGOE

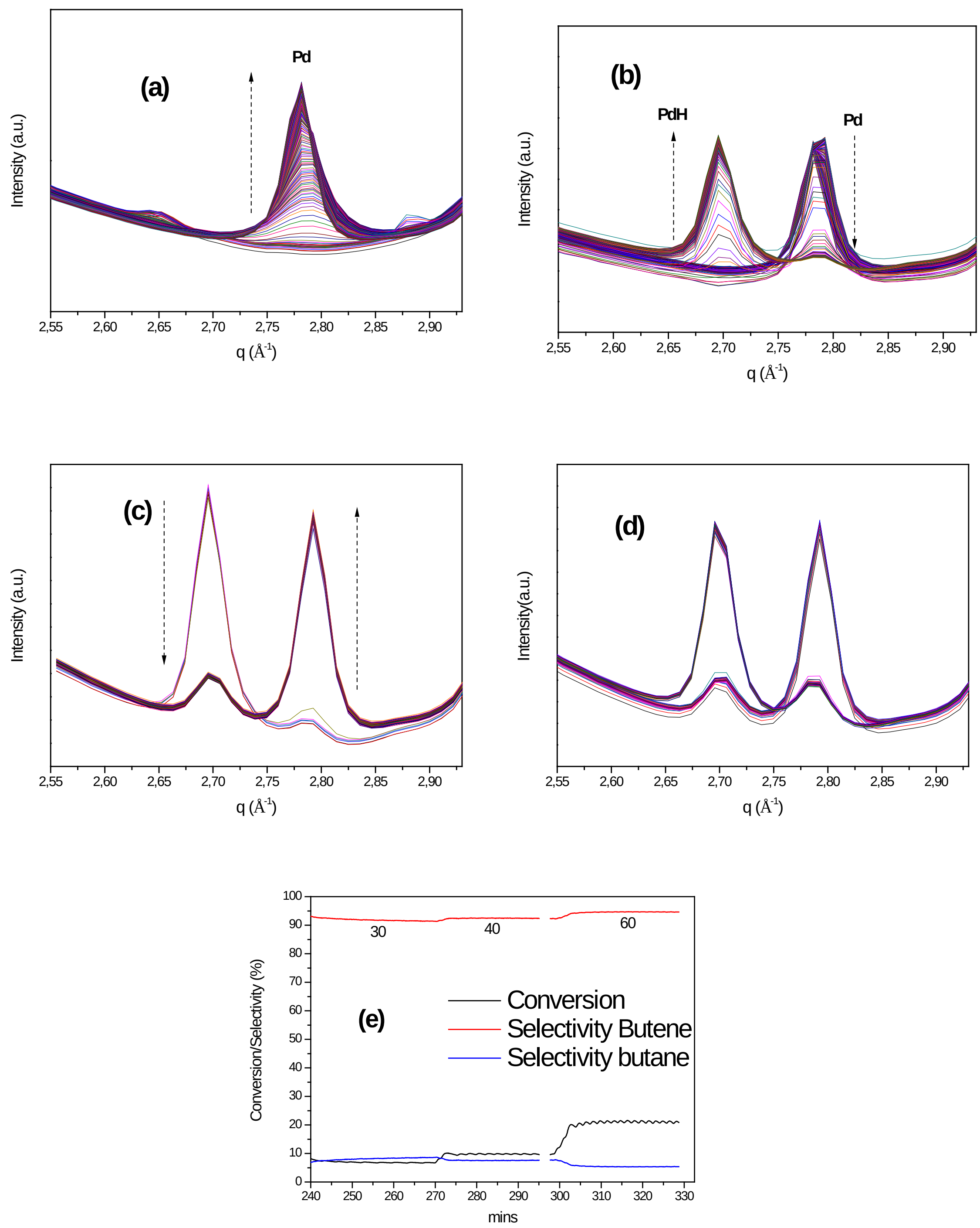

Figure S2. HEXRD of Pd (111) during temperature programmed reduction (TPR) (a), upon cooling (b), temporal evolution on switching from H_2 to reaction mixture during reaction at different temperatures 30 (c), and 60 °C (d) and conversion/selectivity results (e) for sample 1PdGOE.

In this sample, as Figure S2 reveals, the peak due to the formation of Pd nanoparticles (a) during the TPR process is more prominent than for sample 1PdG (Figure S1 (a)). Thereafter, upon cooling, a gradual decrease is observed accompanied by the appearance of a new peak at a lower q value (at about 2.7 Å^{-1}). This corresponds to the transformation of Pd to PdHx species and is almost completed on reaching room temperature (b). Small changes in q values when decreasing temperature can be ascribed to thermal contraction effect in the nanocrystals (e.g. Figure S3 (b)). When this sample is subjected to the studied reaction, the Pd specie is recovered at the expense of the PdHx species ((c)-(d)). As refers to its catalytic behavior, 1PdGOE exhibits much lower conversion values than that obtained with 1PdG, although these values increase with increasing reaction temperature and in all cases it is highly selective towards the partially hydrogenated product, butene (e).

1.3. HEXRD of Pd (111) for sample 1PdGONE

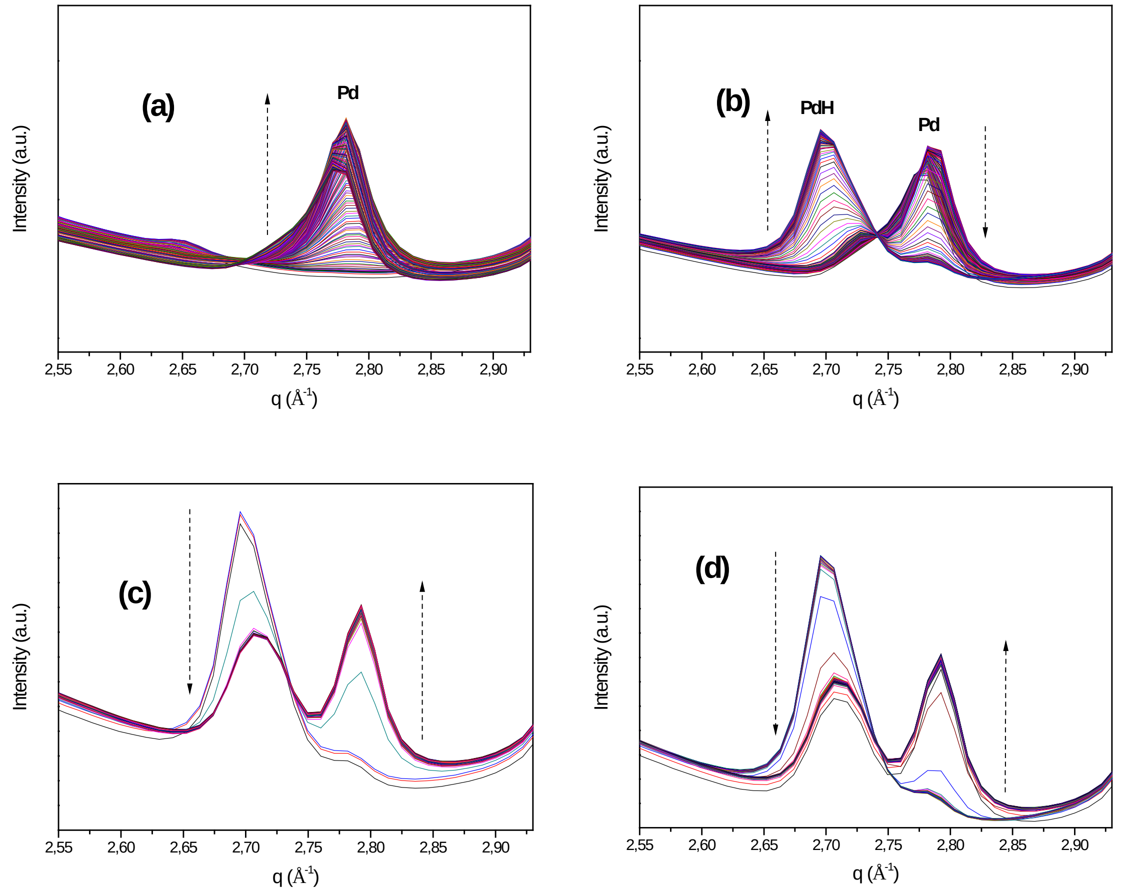

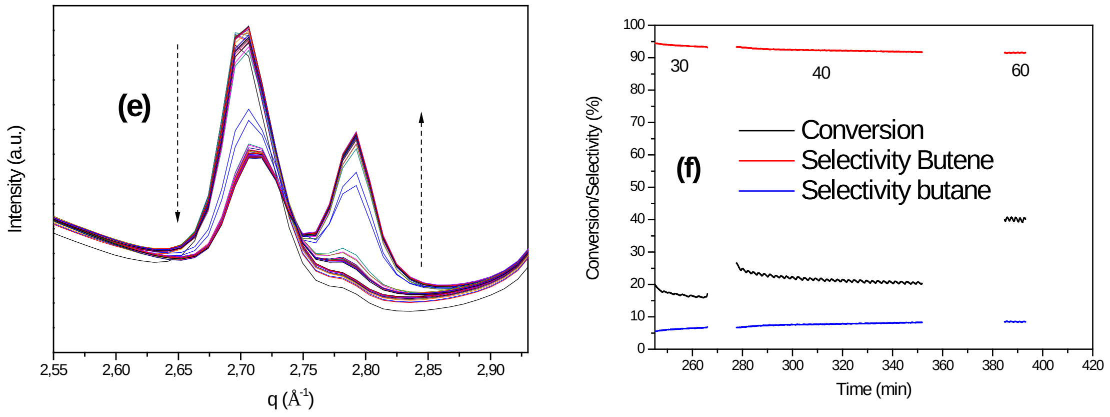

Figure S3. HEXRD of Pd (111) during temperature programmed reduction (TPR) (a), upon cooling (b), temporal evolution on switching from H_2 to reaction mixture during reaction at different temperatures 30 (c), 40 (d) and 60 °C (e) and conversion/selectivity (f) for sample 1PdGONE.

As for 1PdGONE, the results obtained were similar to those observed in 1PdGOE (Figure S2.) although, some differences can be highlighted. In general, peaks seem to be more asymmetric, indicating that the participation of more than one Pd or PdHx specie is more significant in this catalyst. The degree of transformation of PdHx, formed after TPR and cooling under hydrogen, to Pd during the catalytic test ((c), (d) and (e)) is slower for this sample when compared with that of 1PdGOE. This shall be further studied in the next set of experiments in order to get a better understanding of the kinetics. As refers to the catalytic behavior, a similar trend as in 1PdGOE was observed, though conversion is a bit higher and selectivity towards butene is lower.

1.4. HEXRD of Pd (111) for sample 1PdGOE: fast experiment

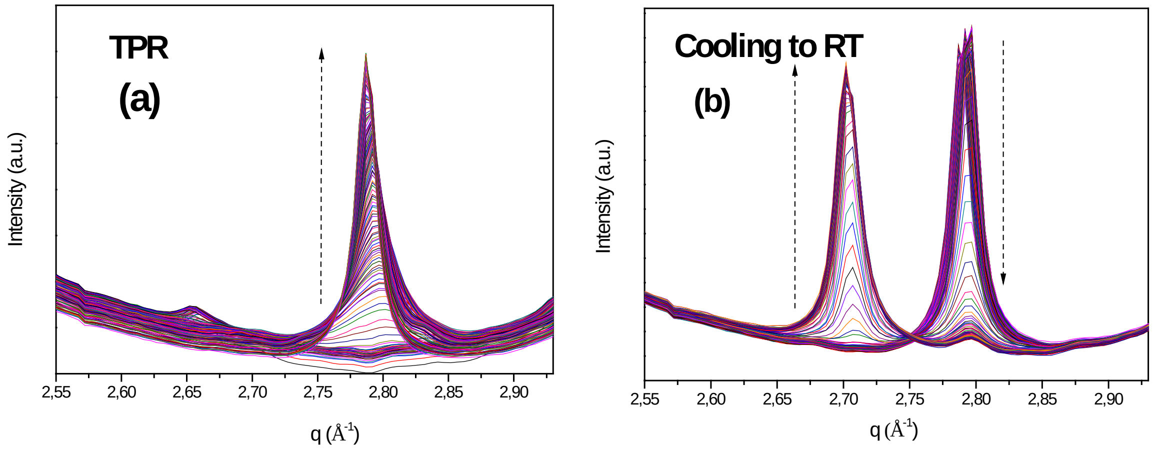

Figure S4. HEXRD of Pd (111) for sample 1PdGOE during temperature programmed reduction (TPR) (a) and upon cooling (b).

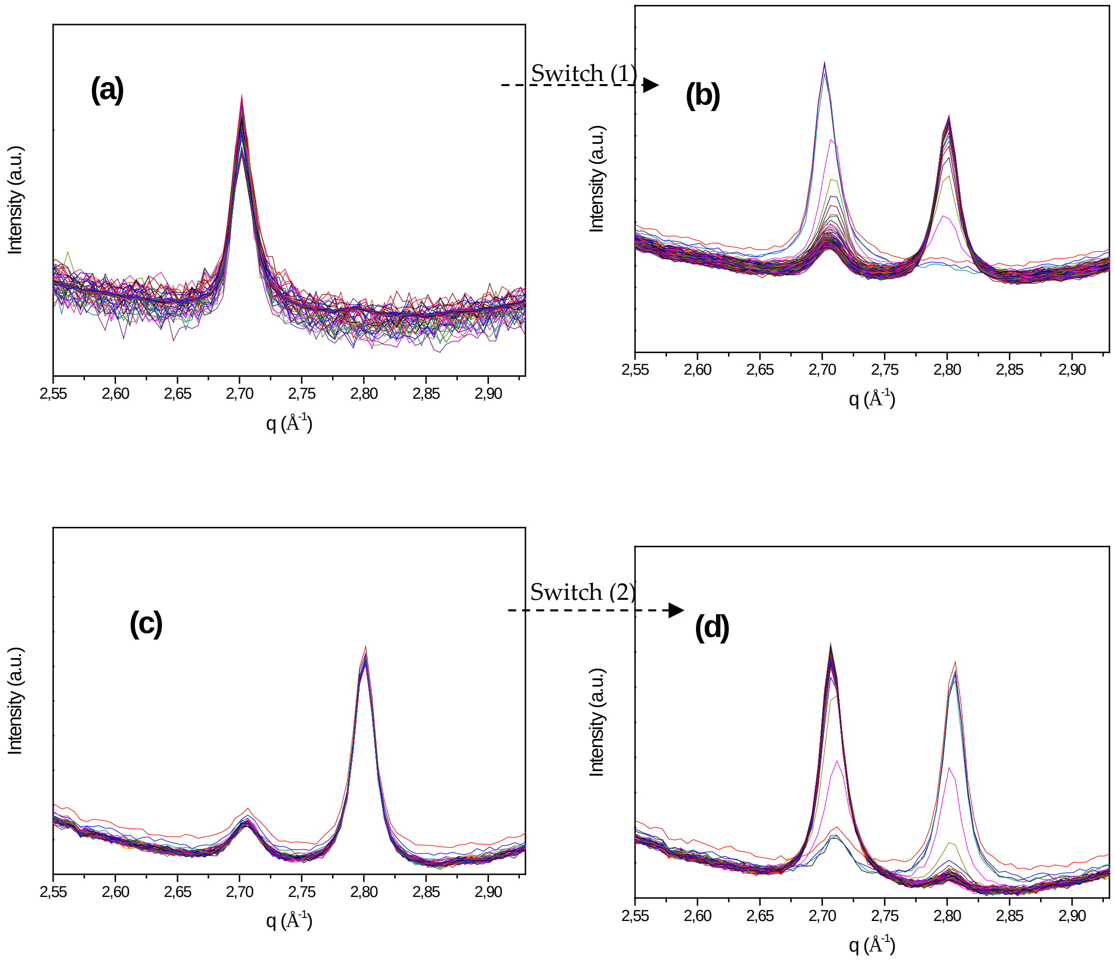

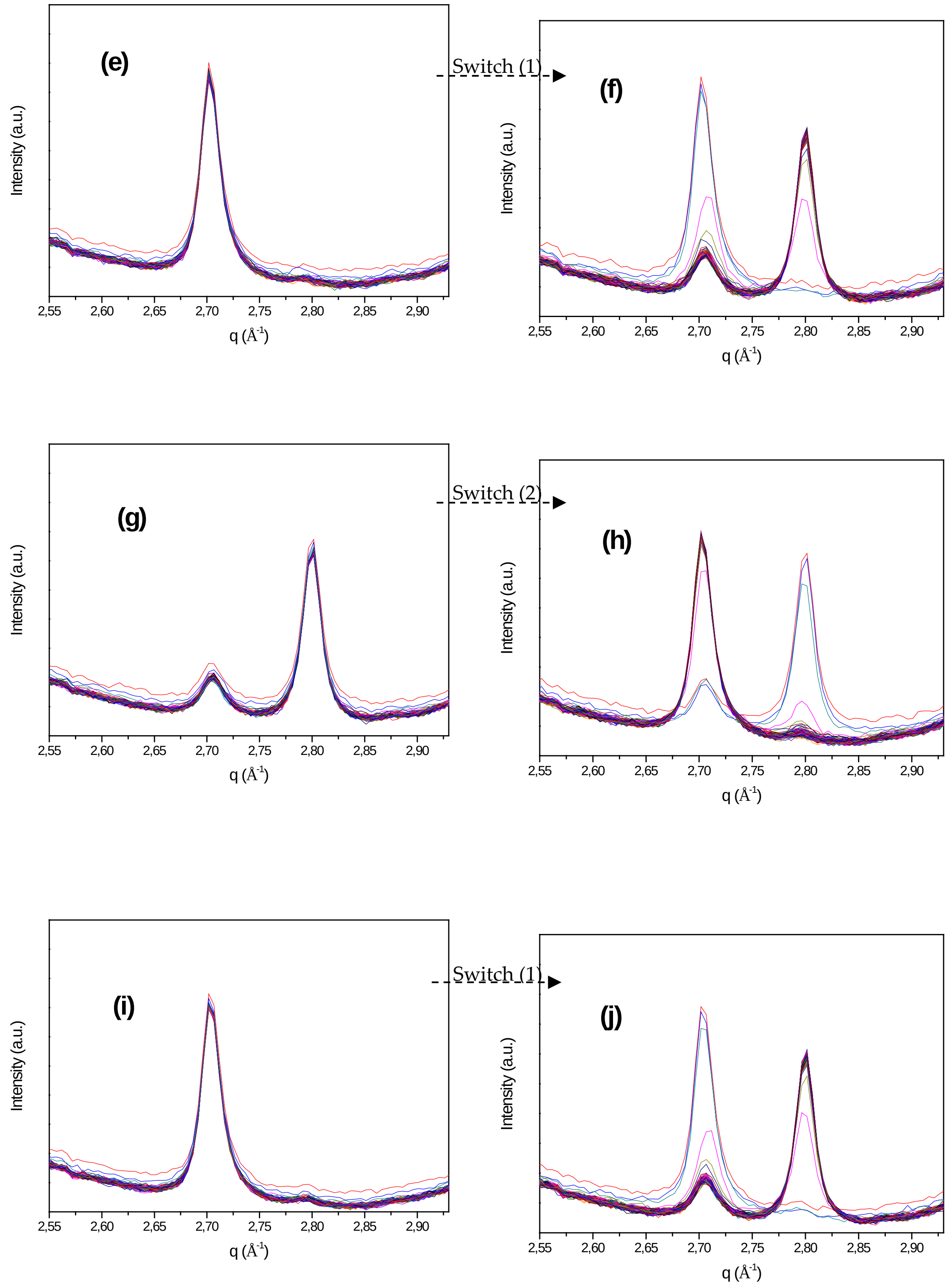
(e)
Switch (1)
(f)
(g)
Switch (2)
(h)
(i)
Switch (1)
(j)
Intensity (a.u.)
q (Å^{-1})
2,55
2,60
2,65
2,70
2,75
2,80
2,85
2,90

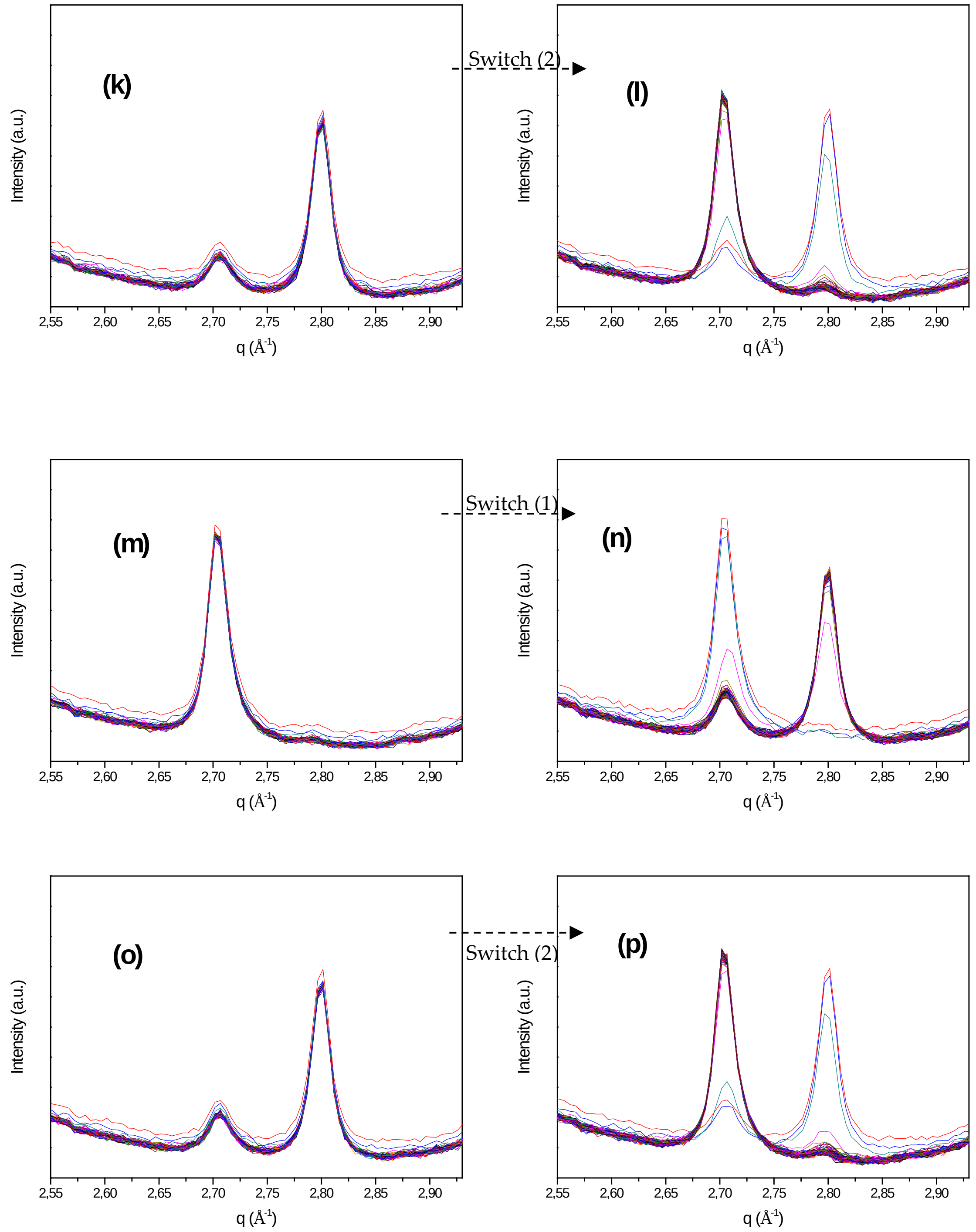
(k)
Switch (2)
(l)
(m)
Switch (1)
(n)
(o)
Switch (2)
(p)
Intensity (a.u.)
q (Å-1)
2,55
2,60
2,65
2,70
2,75
2,80
2,85
2,90

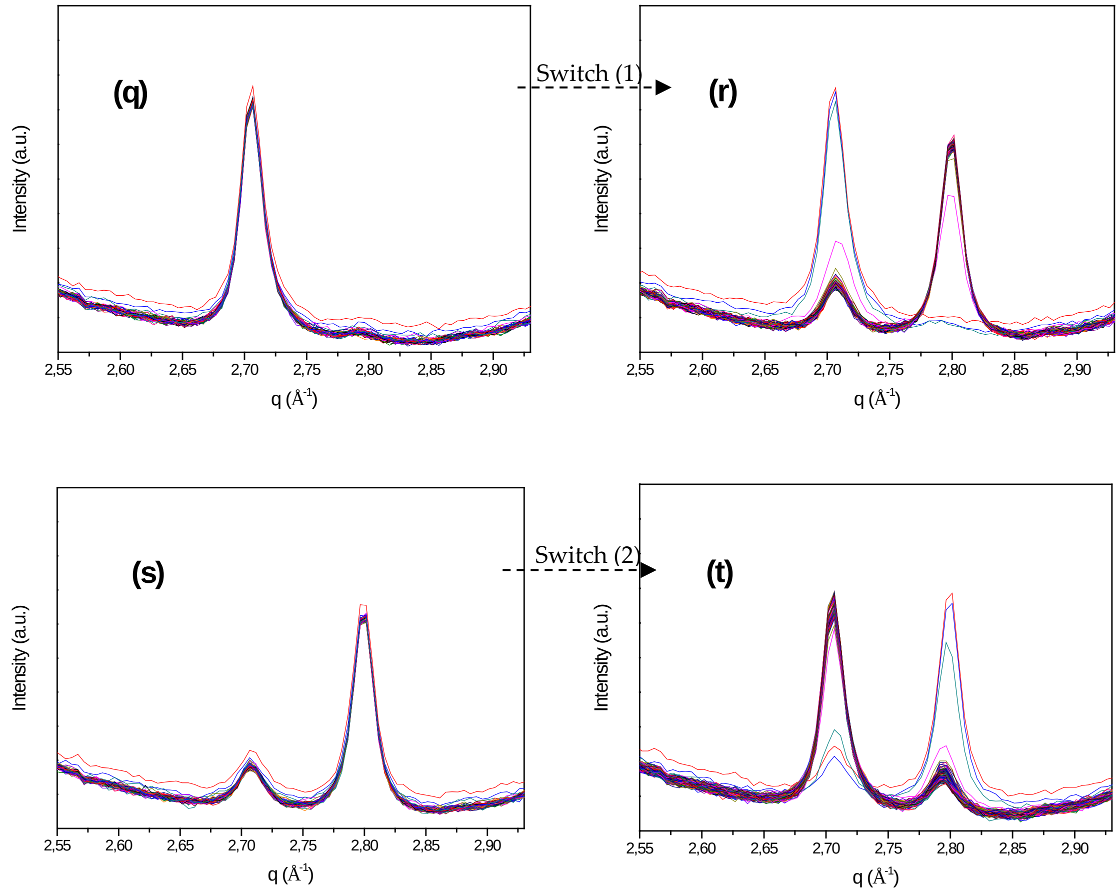

Figure S5. HEXRD of Pd (111) for sample 1PdGOE during reaction at different temperatures: 30 ºC: (a), (b), (c) and (d); 40 °C: (e), (f), (g) and (h); 45 ºC: (i), (j), (k) and (l); 50 °C; (m), (n), (o) and (p); and 60 °C: (q), (r), (s) and (t). Switch (1): Hydrogen to butadiene reaction mixture. Switch (2): butadiene reaction mixture to hydrogen. Figures (a), (c), (e), (g), (l), (k), (m), (o), (q) and (s) correspond to the acquisition during steady state before switching to H_2 or to reaction mixture.

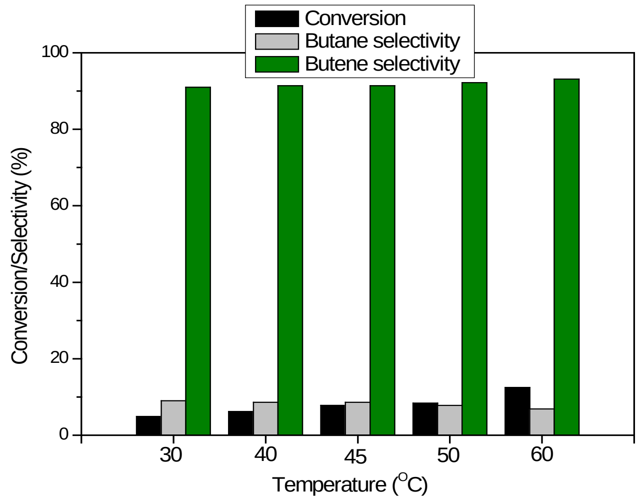

Figure S6. Conversion/selectivity for sample 1PdGOE fast.

In this set of experiments, the acquisition parameters have been varied (read section 1.3. of this supporting information document) in order to follow the transformation of species studied in this work more closely (from Pd to PdHx and vice versa). For all the temperatures studied, the same procedure involving various steps is carried out. We will explain it for just one of the studied temperatures (30 °C). During reduction (TPR) Pd nanoparticles are formed (a) and after cooling to room temperature they are transformed to PdHx (b) as can be seen in Figure S4. and Figure 1 of the main article (both phenomena are also observed in the previous set of reactions with this same sample). The envelop of the PdHx peak could be deconvoluted into two components (α and β hydride species) as will be viewed later on (Figure S10). Prior to introducing the reactive mixture, the sample is kept under hydrogen/helium atmosphere at the reaction temperature (30 °C) for 30 minutes. Hence, the peak observed in (Figure S5 (a)) due to the PdHx present. Thereafter, there is a switch (switch (1)) from the reductive mixture to the reactive mixture (containing butadiene, read section 1.3 of this supporting information for more details) and the difractograms represented in S5(b) illustrate what takes place during the next 30 minutes after switch (1). As can be seen, there is a gradual transformation from PdHx to Pd (owing to the faster acquisition of these experiments higher number of transformation intermediates are observed, in comparison with the other set of reactions, enabling a better follow-up of the temporal evolution). It is also clear that the transformation is not complete as can be viewed in Figure S5 (c) where the sample is kept under the reactive flow before switching (switch (2)) again to reductive flow. Upon switching, diffraction patterns are registered for 30 minutes and the evolution back to PdHx species is observed (Figure S5 (d)). All the above mentioned steps are repeated for the different temperatures studied (from 30 – 60 °C) and their results are collected in figs (e) to (t). Transformation of one Pd specie to another is faster with increasing reaction temperature. Figure S6 depicts conversion and selectivity of the butadiene hydrogenation reaction in the temperature range studied. As was expected and observed in the previous set of experiments, this sample is highly selective to butene formation.

1.5. HEXRD of Pd (111) for sample 1PdGONE fast experiment

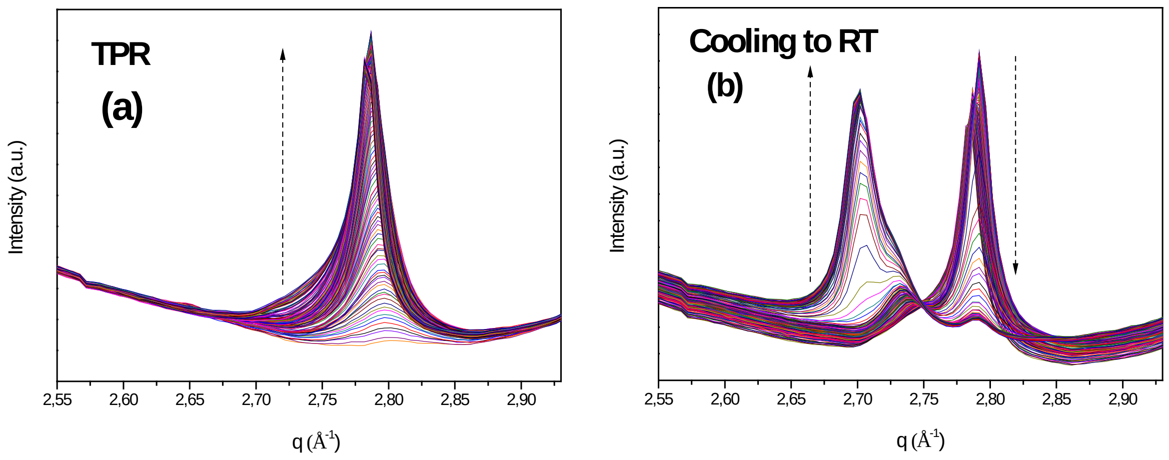

Figure S7. HEXRD of Pd (111) for sample 1PdGONE during temperature programmed reduction (TPR) (a) and upon cooling (b).

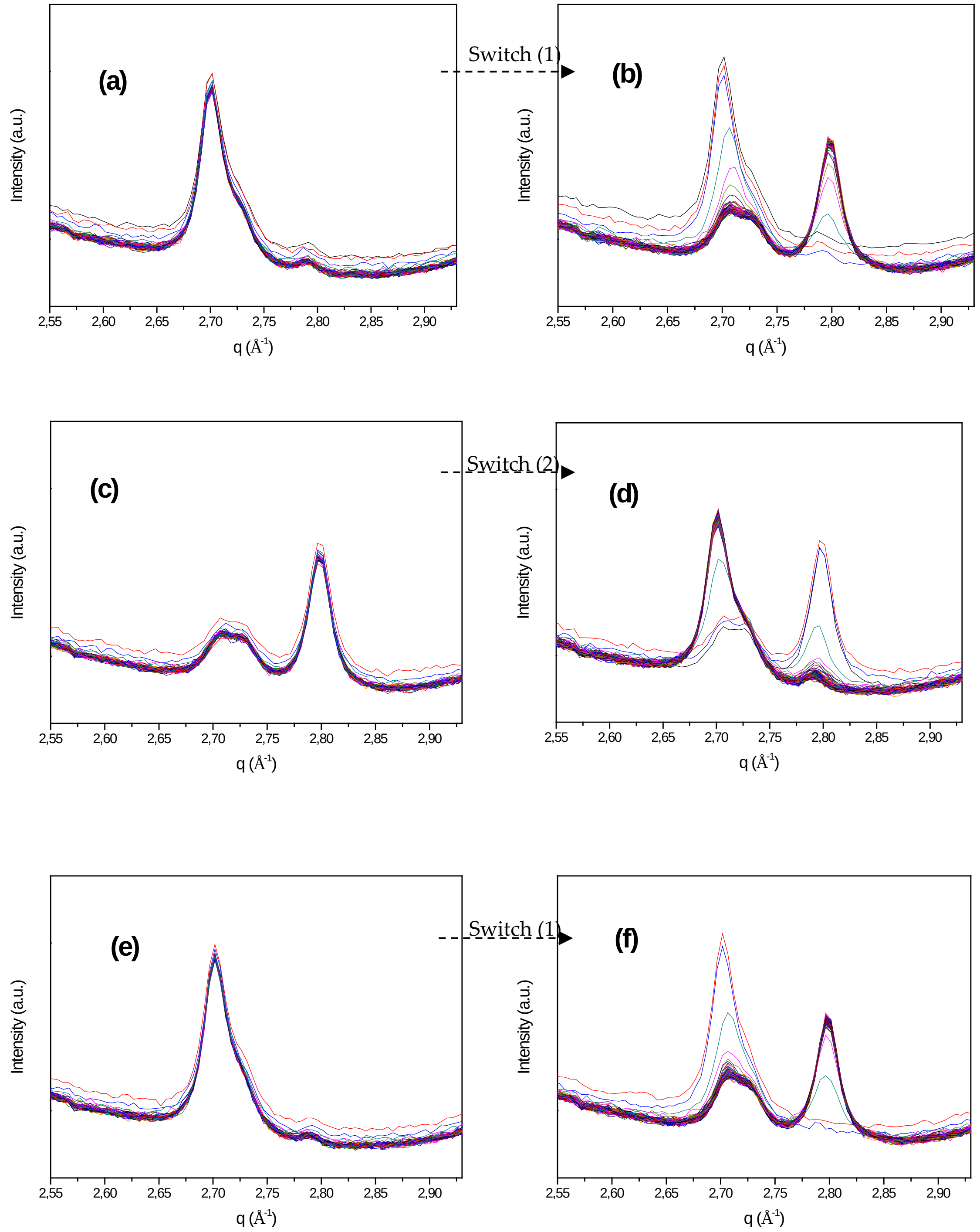

(a)
Switch (1)
(b)
(c)
Switch (2)
(d)
(e)
Switch (1)
(f)
Intensity (a.u.)
q (Å-1)
2,55
2,60
2,65
2,70
2,75
2,80
2,85
2,90

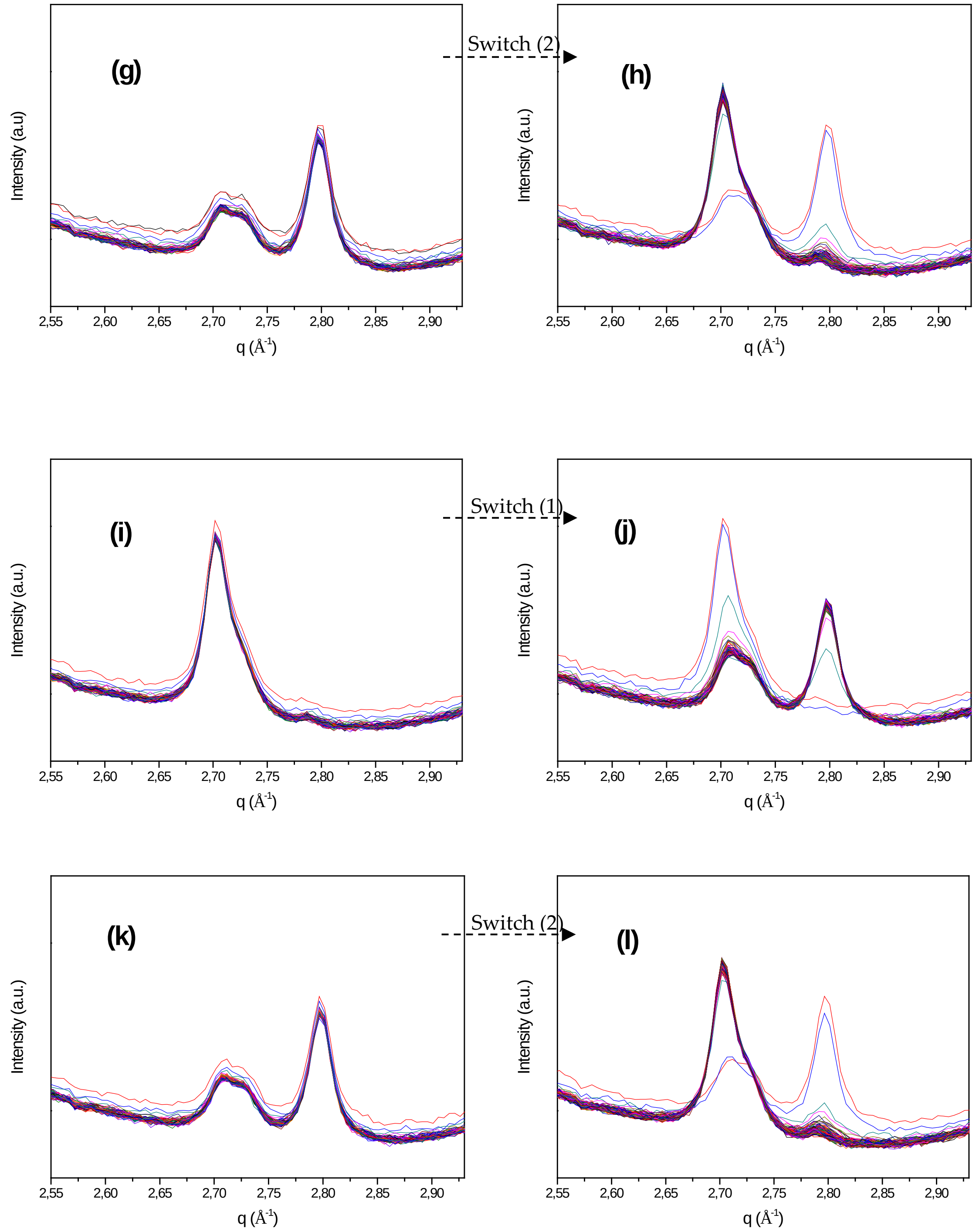

Figure S8. HEXRD of Pd (111) for sample 1PdGONE during reaction at different temperatures: 30 °C: (a), (b), (c) and (d); 40 °C: (e), (f), (g) and (h); and 50°C: (i), (j), (k) and (l). Switch (1): Hydrogen to butadiene reaction mixture. Switch (2): butadiene reaction mixture to Hydrogen. Figures (a), (c), (e), (g), (l) and (k) correspond to the acquisition during steady state before switching to H_2 or to reaction mixture.

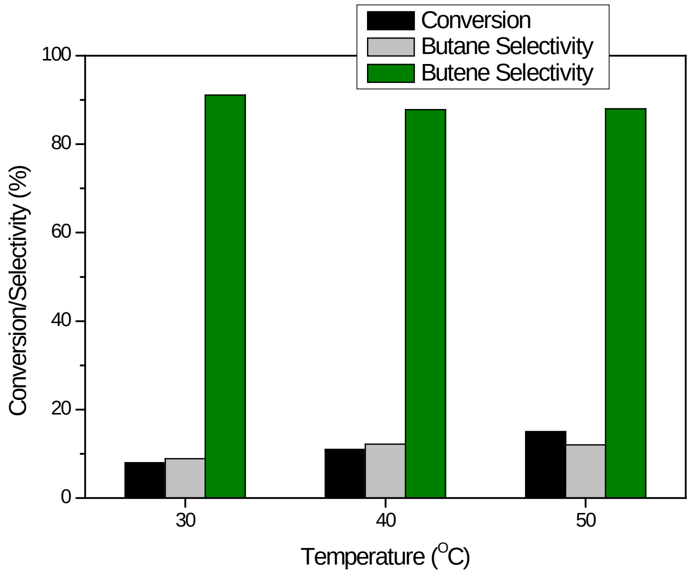

Figure S9. Conversion/selectivity for sample 1PdGONE fast.

The same set of experiments and procedure are performed here as those reported in Figure S4, S5 and S6. In order to avoid being repetitive we would go directly to point out the differences in the results observed between both samples (1PdGOE and 1PdGONE). The participation of more than one specie is more pronounced in this case (compare Figure S4 (b) and S5 (a) with Figure S7 (b) and S8 (a)). Precisely speaking, 1PdGONE has higher contribution of the α component. This can be further highlighted in Figure S10 (a) and (b) where a deconvolution of envelops of the PdHx peak formed after TPR and cooling to RT is presented. The difference in the participation of the α component is quite significant. At the same time, the transformation of PdHx to Pd is less complete for this sample (83% vs. 66%). This is probably due to the higher contribution of the α PdHx which seems to participate less in the transformation as can be viewed in these difractograms (view also Figure S8 (b)). The deconvolution of the envelops of peaks after transformation of PdHx to Pd also confirms a loss of the β component during the process owing to its higher participation (Figure S10 (c) and (d)). As for the conversion and selectivity results presented in Figure S9, these are quite similar, as was expected, to those observed in Figure S6.

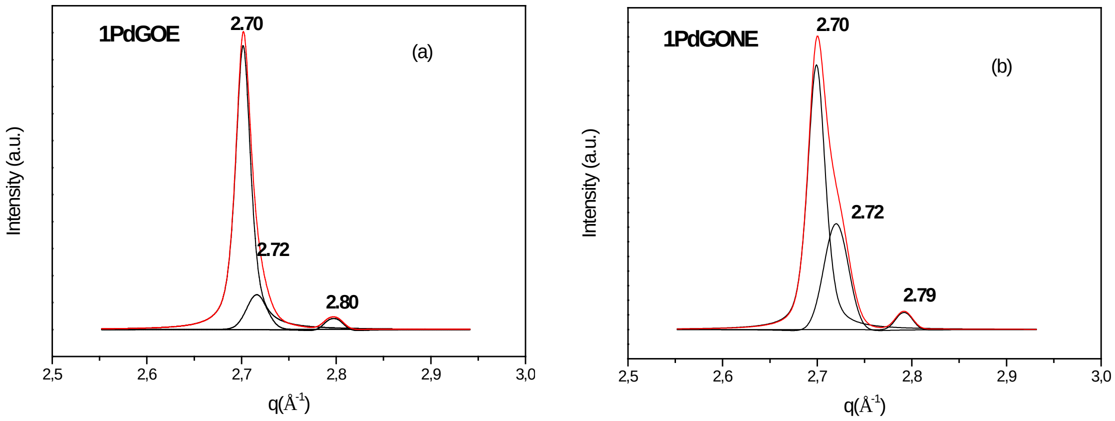

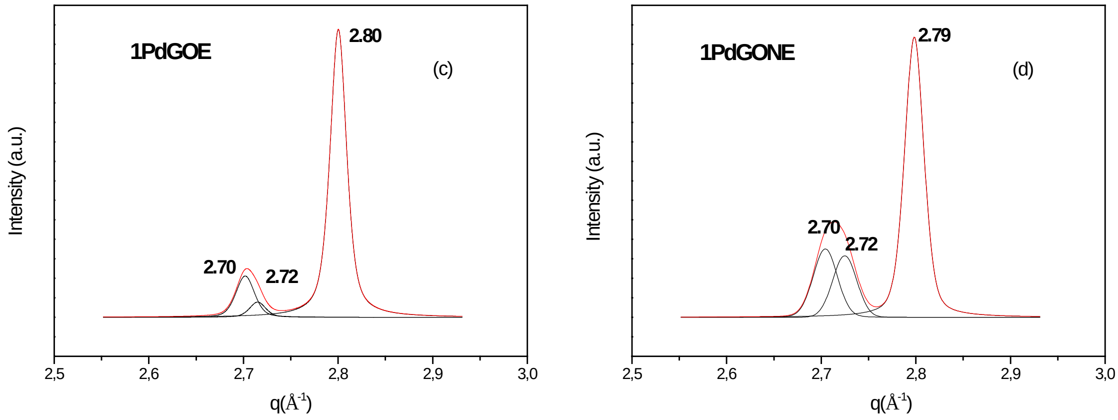

Figure S10. Deconvolution of PdHx peak for 1PdGOE and for 1PdGONE samples under H_2 atmosphere ((a) and (b)) and under reaction mixture conditions ((c) and (d)).

1.6. HEXRD of Pd (111) for sample 2PdG fast

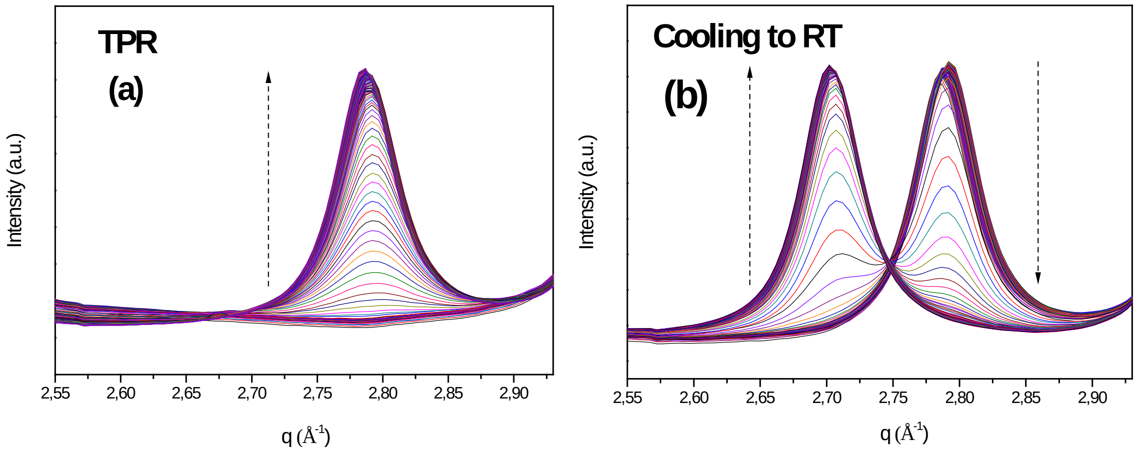

Figure S11. HEXRD of Pd (111) for sample 2PdG during temperature programmed reduction (TPR) (a) and upon cooling (b).

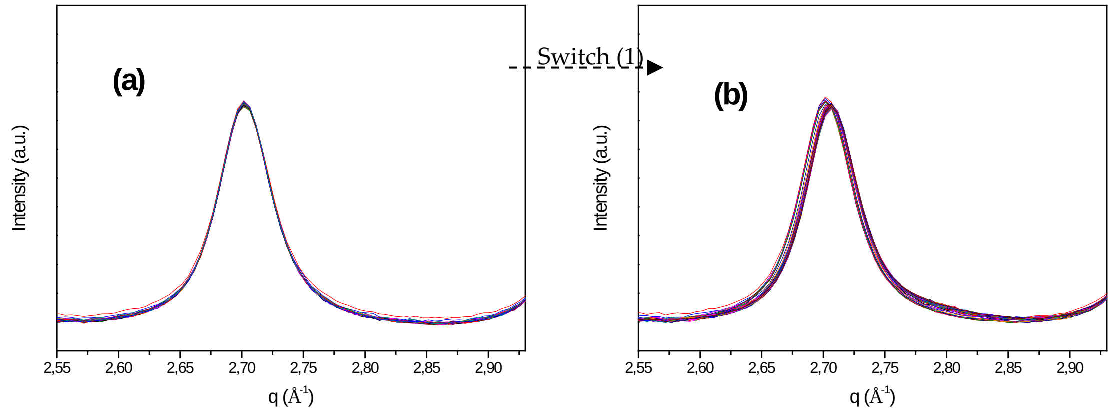

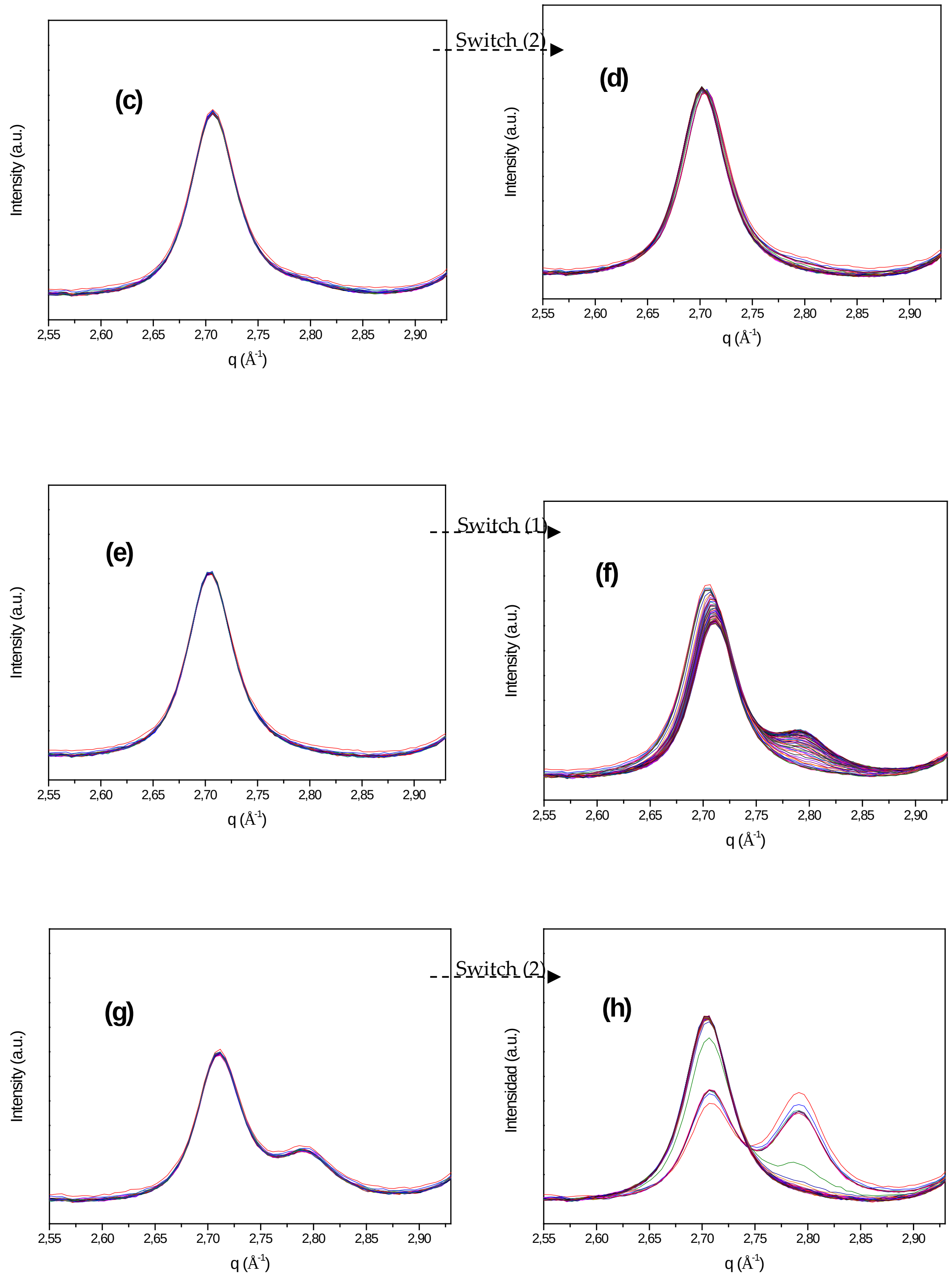
(c)
Intensity (a.u.)
2,55 2,60 2,65 2,70 2,75 2,80 2,85 2,90
q (Å-1)
Switch (2)
(d)
Intensity (a.u.)
2,55 2,60 2,65 2,70 2,75 2,80 2,85 2,90
q (Å-1)
(e)
Intensity (a.u.)
2,55 2,60 2,65 2,70 2,75 2,80 2,85 2,90
q (Å-1)
Switch (1)
(f)
Intensity (a.u.)
2,55 2,60 2,65 2,70 2,75 2,80 2,85 2,90
q (Å-1)
(g)
Intensity (a.u.)
2,55 2,60 2,65 2,70 2,75 2,80 2,85 2,90
q (Å-1)
Switch (2)
(h)
Intensidad (a.u.)
2,55 2,60 2,65 2,70 2,75 2,80 2,85 2,90
q (Å-1)

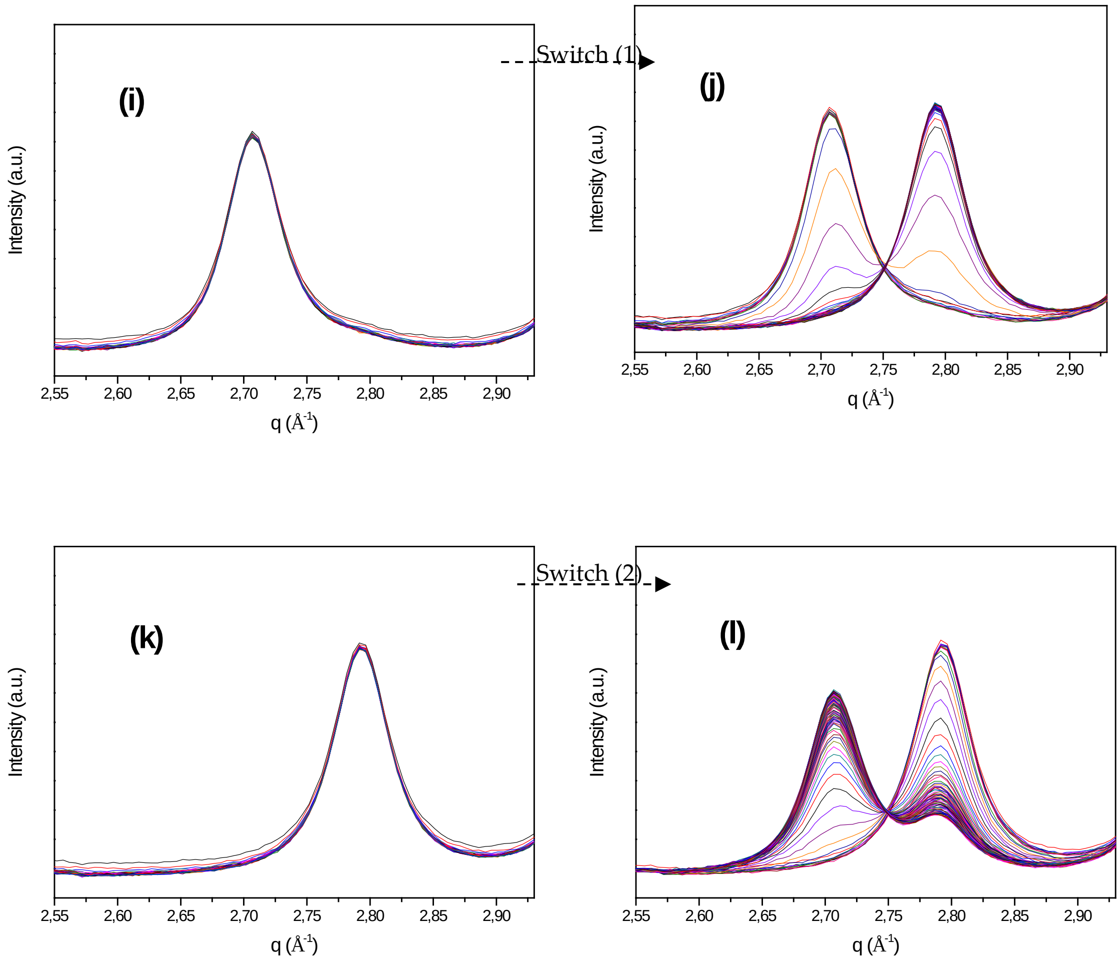

Figure S12. HEXRD of Pd (111) for sample 2PdG during reaction at different temperatures: 30 °C: (a), (b), (c) and (d); 50 °C: (e), (f), (g) and (h); and 70°C: (i), (j), (k) and (l). Switch (1): Hydrogen to butadiene reaction mixture. Switch (2): butadiene reaction mixture to Hydrogen.Figures (a), (c), (e), (g), (l) and (k) correspond to the acquisition during steady state before switching o H_2 or to reaction mixture.

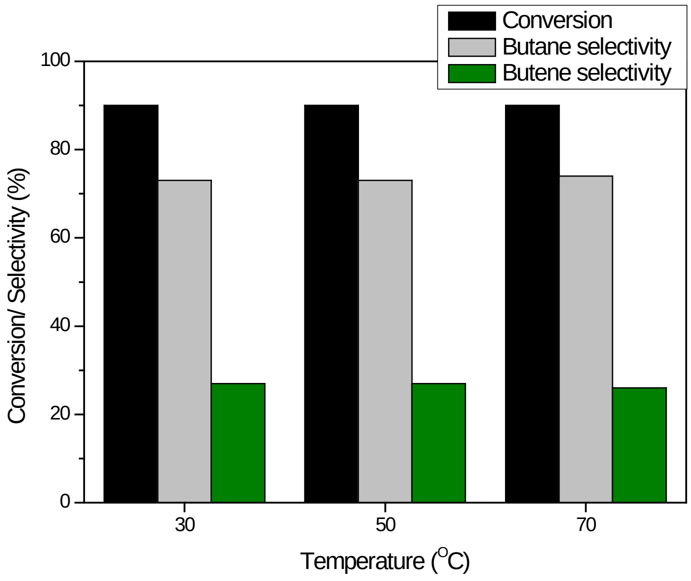

Figure S13. Conversion/selectivity for sample 2PdG fast.

2PdG exerted a very different behavior. On one hand, comparing with 1PdG (also supported on graphite), a more prominent Pd (111) peak was observed confirming that particle size enlargement was achieved. These sizes will later be verified by TEM in Figure S16 and S17. On the other hand, when comparing with 1PdGOE and 1PdGONE, also studied in this set for experiments, clear differences are seen. To start with, in this case peaks are quite symmetric. This could be due to the presence of just one contribution of Pd and PdHx or that the differences between α and maxima have been narrowed to the point where they are coincident. On the other hand, it has been reported in the literature (as collected in the main article) that for small nanoparticle sizes no β PdHx is formed. Hence, in our case we are most likely to have just the α component. Figs. S12 (a), (b), (c) and (d) are identical, all belonging to the difractogram of the PdHx specie formed. This indicates that at temperatures as low as 30°C on switching from reductive mixture to reactive mixture and vice versa there is no transformation to Pd species. At 50°C this transformation is present although to a very low extent. We can compare this behavior to that observed for the other two samples, all three studied at 50°C. 1PdGOE was about 83%, 1PdGONE was 66% while 2PdG was only 22%. These differences could be based on their different particle sizes as can be viewed in the next figures (S14- S16) where TEM images are illustrated. At higher temperatures (70°C) transformation was slow but complete. High conversions and low selectivity towards butenes were attained with this catalyst.

1.7. TEM images and particle size histogram of 1PdGOE

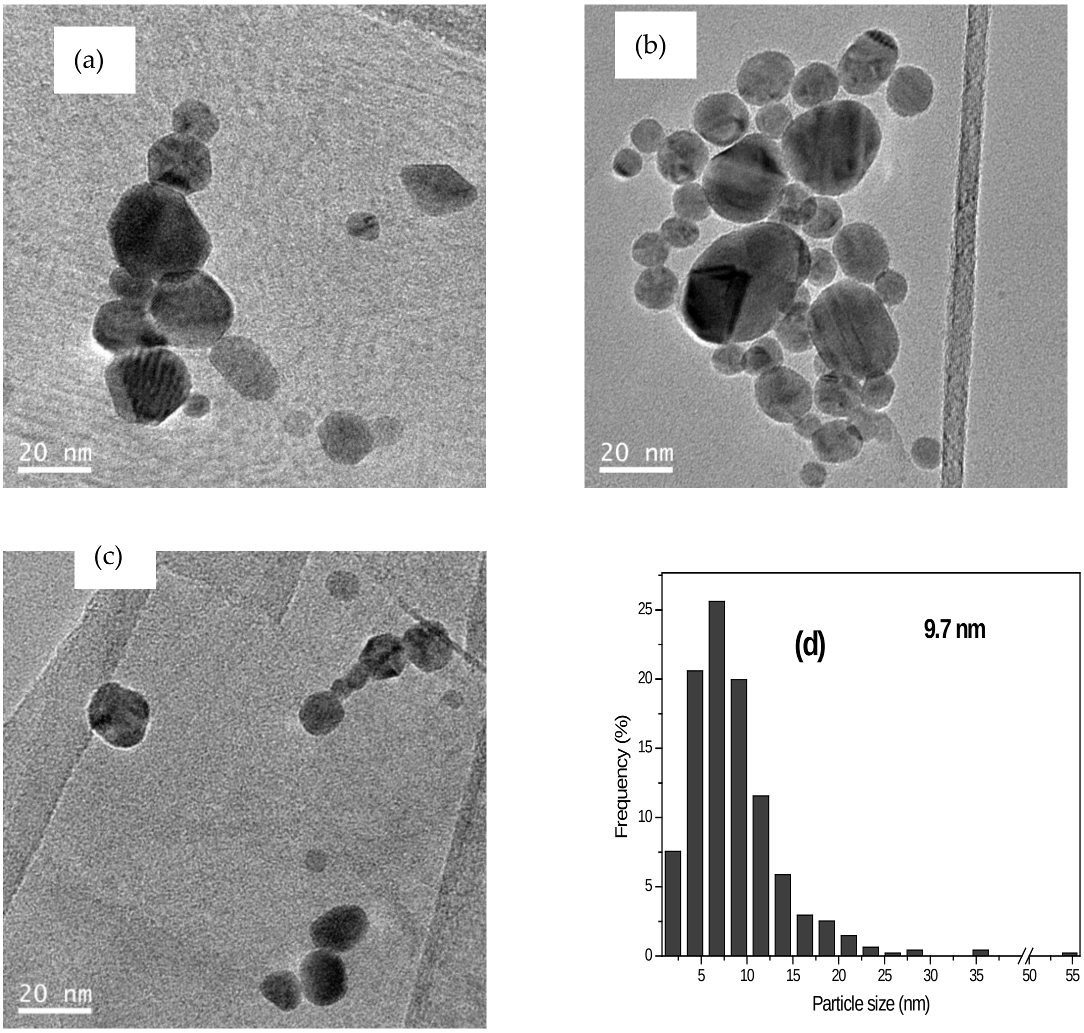

Figure S14. TEM images ((a), (b) and (c)) and particle size histogram (d) of 1PdGOE.

1.8. TEM images and particle size histogram of 1PdGONE

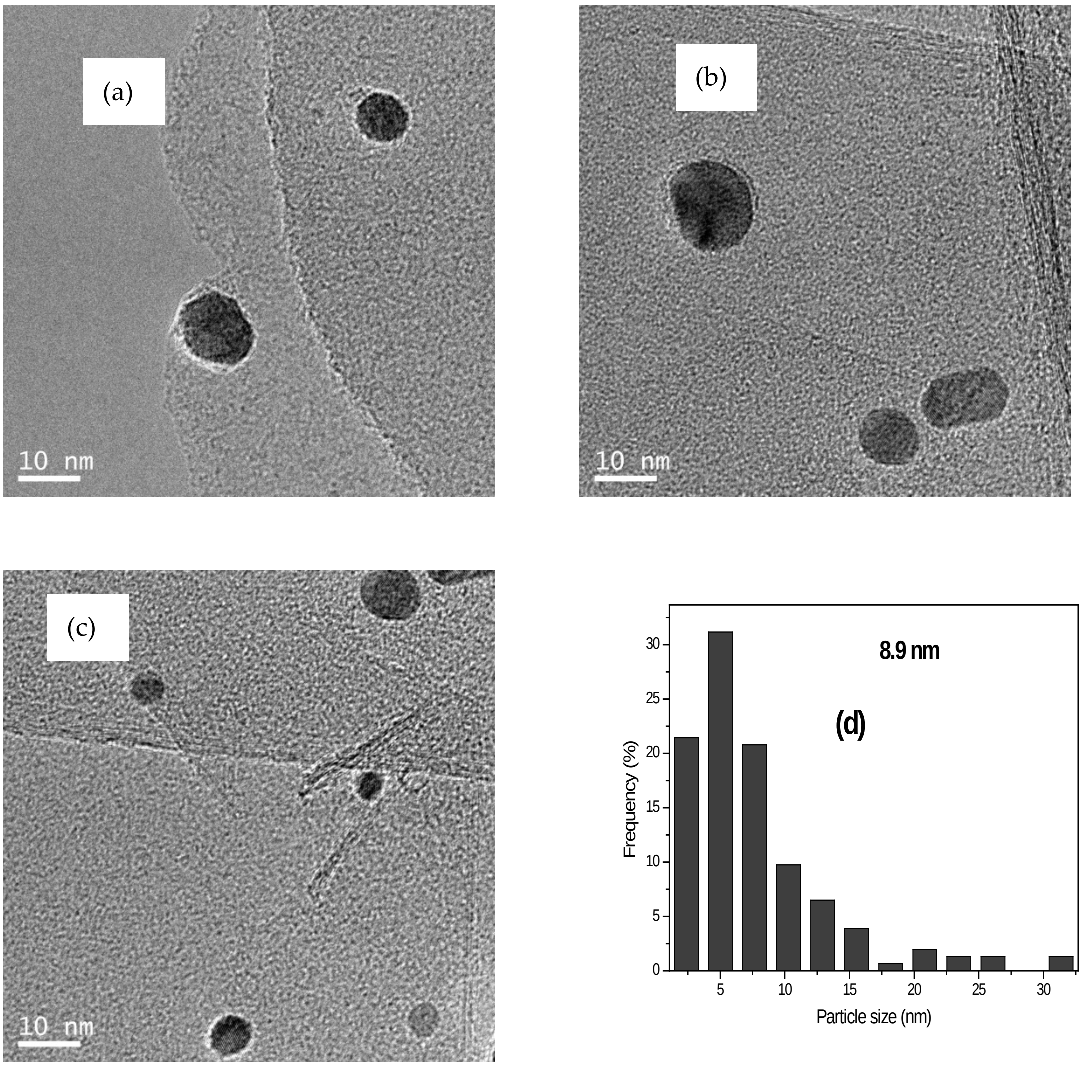

Figure S15. TEM images ((a), (b) and (c)) and particle size histogram (d) of 1PdGONE.

1.9. TEM images and particle size histogram of 2PdG

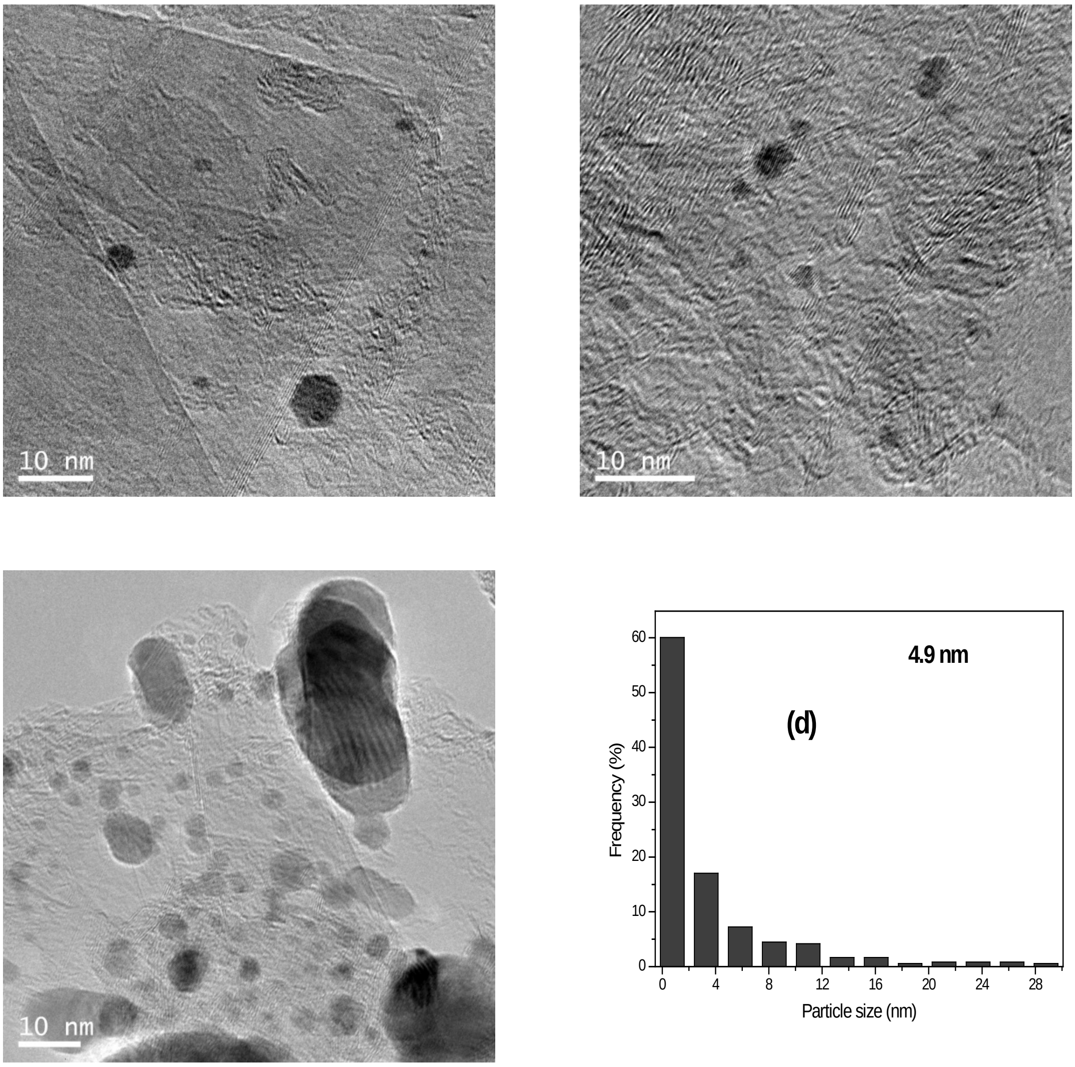

Figure S16. TEM images ((a), (b) and (c)) and particle size histogram (d) of 2PdG.

1.10. TEM images and particle size histogram of 1PdG

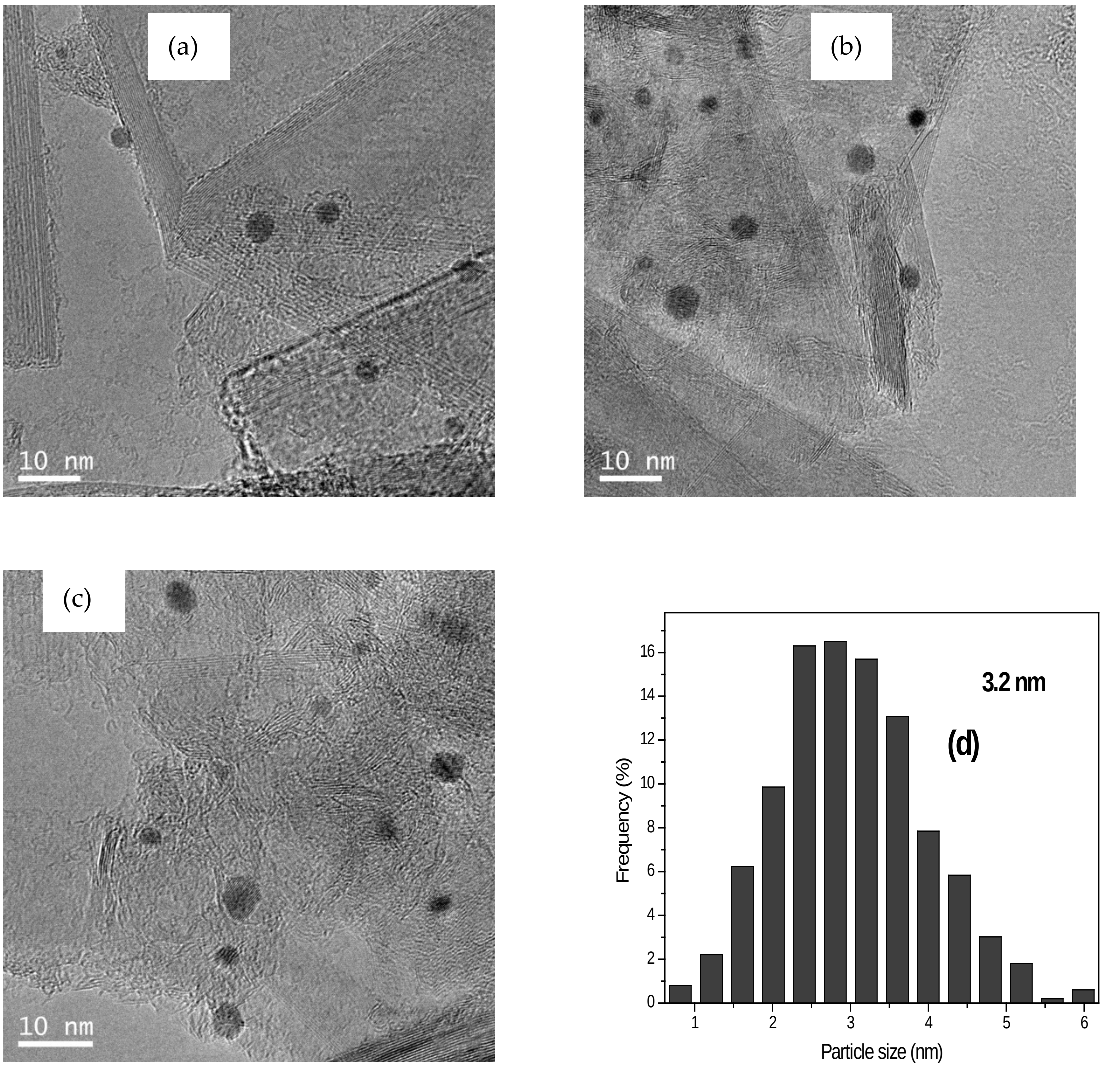

Figure S17. TEM images ((a), (b) and (c)) and particle size histogram (d) of 1PdG.

1.11. Kinetics of transformation of PdH_x to Pd

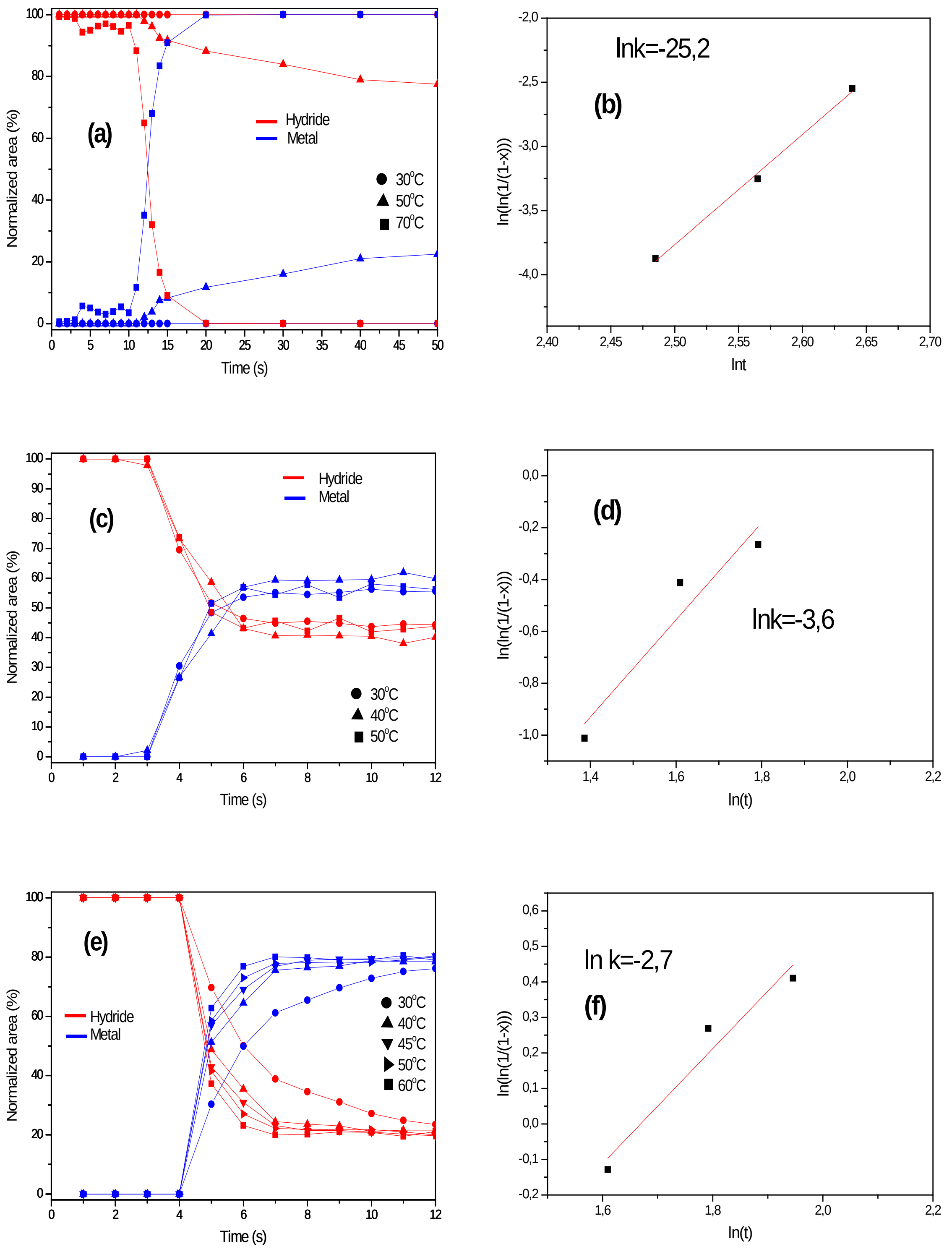

Figure S18. Kinetics of transformation of PdHx to Pd: 2PdG (a) and (b), 1PdGONE (c) and (d), 1PdGOE (e) and (f).

Presenting normalized areas of diffraction peaks as a function of the transformation time (from PdHx to Pd), (a), (c) and (e) are obtained for 2PdG, 1PdGONE and 1PdGOE, respectively, at the different reaction temperatures studied. All three summarize the results discussed in previous figures. At the same time, employing the Avrami model, the transformation rate constant (K) was deduced for all samples at the same reaction temperature (50 °C). This value is highest for 1PdGOE and lowest for 2PdG and these results follow the same tendency as that observed for the selectivity towards the partially hydrogenated product, butene, which requires a fast transformation process of β PdHx into Pd.
